# Diet Composition and Variability of Wild *Octopus vulgaris* and *Alloteuthis media* (Cephalopoda) Paralarvae: a Metagenomic Approach

**DOI:** 10.3389/fphys.2017.00321

**Published:** 2017-05-24

**Authors:** Lorena Olmos-Pérez, Álvaro Roura, Graham J. Pierce, Stéphane Boyer, Ángel F. González

**Affiliations:** ^1^Instituto de Investigaciones Marinas, Ecobiomar, CSICVigo, Spain; ^2^La Trobe UniversityMelbourne, VIC, Australia; ^3^CESAM and Departamento de Biologia, Universidade de AveiroAveiro, Portugal; ^4^Applied Molecular Solutions Research Group, Environmental and Animal Sciences, Unitec Institute of TechnologyAuckland, New Zealand

**Keywords:** trophic ecology, NW Iberian Peninsula, paralarvae culture, NGS diet analysis, Illumina Miseq

## Abstract

The high mortality of cephalopod early stages is the main bottleneck to grow them from paralarvae to adults in culture conditions, probably because the inadequacy of the diet that results in malnutrition. Since visual analysis of digestive tract contents of paralarvae provides little evidence of diet composition, the use of molecular tools, particularly next generation sequencing (NGS) platforms, offers an alternative to understand prey preferences and nutrient requirements of wild paralarvae. In this work, we aimed to determine the diet of paralarvae of the loliginid squid *Alloteuthis media* and to enhance the knowledge of the diet of recently hatched *Octopus vulgaris* paralarvae collected in different areas and seasons in an upwelling area (NW Spain). DNA from the dissected digestive glands of 32 *A. media* and 64 *O. vulgaris* paralarvae was amplified with universal primers for the mitochondrial gene COI, and specific primers targeting the mitochondrial gene 16S gene of arthropods and the mitochondrial gene 16S of Chordata. Following high-throughput DNA sequencing with the MiSeq run (Illumina), up to 4,124,464 reads were obtained and 234,090 reads of prey were successfully identified in 96.87 and 81.25% of octopus and squid paralarvae, respectively. Overall, we identified 122 Molecular Taxonomic Units (MOTUs) belonging to several taxa of decapods, copepods, euphausiids, amphipods, echinoderms, molluscs, and hydroids. Redundancy analysis (RDA) showed seasonal and spatial variability in the diet of *O. vulgaris* and spatial variability in *A. media* diet. General Additive Models (GAM) of the most frequently detected prey families of *O. vulgaris* revealed seasonal variability of the presence of copepods (family Paracalanidae) and ophiuroids (family Euryalidae), spatial variability in presence of crabs (family Pilumnidae) and preference in small individual octopus paralarvae for cladocerans (family Sididae) and ophiuroids. No statistically significant variation in the occurrences of the most frequently identified families was revealed in *A. media*. Overall, these results provide new clues about dietary preferences of wild cephalopod paralarvae, thus opening up new scenarios for research on trophic ecology and digestive physiology under controlled conditions.

## Introduction

Historically, cephalopods in European waters have always been viewed as a minor fisheries resource (Pierce et al., 2010). However, they can be of considerable local economic importance, especially in southern Europe's artisanal fisheries. Galician waters (NW Spain) support an economically important cephalopod fishery for *Octopus vulgaris* (Otero et al., 2006; Pita et al., 2016) and loliginid squid, mainly *Loligo vulgaris* but also *Alloteuthis media* and *Alloteuthis subulata* (Jereb et al., 2015), species that are not easily distinguished due to the similarity of their external characters (Jereb et al., 2010). Reflecting the short life cycle and rapid individual growth rates, cephalopod populations are sensitive to effects of environmental variation on reproduction and recruitment (Boyle, 1990; Boyle and Rodhouse, 2005; Pierce et al., 2008; Hastie et al., 2009; Rodhouse et al., 2014), resulting in wide year to year fluctuations in captures. From 2000 to 2013, reported cephalopod landings in Europe varied from a minimum of 38,600 tons in 2009 to a maximum of 55,500 tons in 2004 (ICES, 2014).

In recent years, there has been growing interest in the culture of cephalopods, primarily for human consumption, due to their high growth rates, high protein contents, and high ratios of food conversion and short life cycles (Segawa, 1990; Lee, 1995; Villanueva and Bustamante, 2006). Bearing in mind the variability of the wild cephalopod resources, there is a need for a stable and reliable source of cephalopods. Additional impetus for captive rearing arises from the use of cephalopods as model organisms in biomedical science (Bullock, 1948; Hanlon, 1990; Fiorito and Scotto, 1992; Calisti et al., 2011) and for ornamental purposes (Dunstan et al., 2010; Rodhouse et al., 2014). Despite progress in cephalopod culture methods (e.g., Iglesias et al., 2014), cephalopod species with planktonic stages have very low survival rates of paralarvae in captive conditions (Villanueva and Norman, 2008). Therefore, rearing relies on wild captured juveniles, subadults, and adults, preventing commercial viability (Hernández Moresino et al., 2014; Xavier et al., 2015).

Juvenile and sub-adult cephalopods are mainly fed with live prey, including crustaceans, fishes, and mollusks (Domingues et al., 2004; García García and Cerezo Valverde, 2006; Sykes et al., 2013), fisheries discards (Socorro et al., 2005; Estefanell et al., 2011), frozen prey (Ferreira et al., 2010; Sykes et al., 2013), or artificial feed stuffs (Garcia et al., 2011; Estefanell et al., 2013). Paralarvae in captivity have been traditionally fed with enriched *Artemia* (Iglesias et al., 2006) or supplemented with decapod zoeae, copepods, mysids, shrimps, or fish larvae, which increases survival and growth rates (Hernández-García et al., 2000; Iglesias et al., 2004, 2006; Ikeda et al., 2005; Carrasco et al., 2006; Kurihara et al., 2006; Martínez et al., 2014; Farías et al., 2015). Despite all previous attempts, cephalopod paralarval mortality is still close to 100% in captivity.

It has been suggested that the high mortality of paralarvae in captivity is due to a lack of knowledge regarding the physiology and nutrition of paralarvae (Domingues et al., 2003; Villanueva et al., 2009; Garrido et al., 2016a) or the lack of a suitable diet meeting all micronutrient requirements (Iglesias et al., 2007). Several experiments have shown that feeding new born paralarvae with different diet can influence its survival (Villanueva, 1994; Iglesias et al., 2004; Farías et al., 2015). Moreover, physiological changes in digestive gland lipid composition (Garcia et al., 2011) and higher proteolytic activity were observed in paralarvae fed on other zooplankton organisms, rather than Artemia (Pereda et al., 2009).

Moreover, laboratory experiments have shown that hatchlings present restricted swimming capacity and prey hunting skills. They progressively develop the ability to capture different zooplankton prey (Hanlon, 1990; Chen et al., 1996), suggesting the necessity to adapt their diet at their different stages. Thus, increasing the knowledge of dietary preferences of wild cephalopod paralarvae and ontogenetic dietary changes over the course of their early development could help to design a suitable diet for rearing in captivity.

A few investigations have analyzed the diet of wild paralarvae by visual identification of stomach contents, revealing that they mainly fed on copepods (*Illex argentinus*, Vidal and Haimovici, 1998), amphipods (*Ommastrephes bartramii*, Uchikawa et al., 2009), and other crustaceans (*Abralia trigonura* and *Sthenoteuthis oualaniensis*; Vecchione, 1991). However, a high proportion of stomach contents comprises unrecognizable soft material (Roura et al., 2012; Camarillo-Coop et al., 2013) or small pieces of exoskeleton (Passarella and Hopkins, 1991; Vecchione, 1991; Vidal and Haimovici, 1999). Alternative approaches have also been attempted: Specific prey species of *Loligo reynaudii* were detected by applying immunoassays (Venter et al., 1999) and in *O. vulgaris* paralarvae up to 20 different prey were detected cloning PCR products with group-specific primers (Roura et al., 2012). However, these methods are costly and time-consuming, and thus can only be applied to a limited number of samples and clones sequenced.

Zooplankton communities in the Ría de Vigo (NW Iberian Peninsula) are highly dynamic, presenting rapid changes in species composition and abundance according to environmental conditions (Roura et al., 2013; Buttay et al., 2015). Previous research suggests that *O. vulgaris* paralarvae are specialist predators, eating decapods independently of the zooplankton communities they inhabit (Roura et al., 2016). However, a specialist diet focusing on low abundance prey could lead to starvation and death. It is therefore expected that cephalopod paralarvae have certain degree of plasticity in terms of the different prey they can capture, based on their hunting abilities, and that they also eat prey species that have not been yet detected in their diet.

The development of next generation sequencing (NGS) has permitted the elucidation of the diet of a wide variety of animal species including vertebrates and invertebrates (King et al., 2008; Boyer et al., 2013; Leray et al., 2013b). These techniques are more efficient and, in many cases, less costly than traditional diet analysis in terms of time and prey species resolution (Pompanon et al., 2012). Thus, NGS could be applied to reveal previously undetected prey species of cephalopod paralarvae and to extend dietary analysis to a higher number of paralarvae.

Therefore, the aim of this study was to develop a NGS approach to provide a detailed analysis of the diet of the paralarvae of the two cephalopod species most abundant in the plankton in NW Iberian Peninsula. First of all, we describe for the first time the diet of paralarvae of *Alloteuthis media*, and secondly, we present new information on the dietary preferences of paralarvae of *O. vulgaris* in the coastal environment. Environmental conditions (such as season and feeding area) affecting paralarval prey preferences are also assessed. Diet is thought to be the main factor affecting paralarval survival and determining diet is an essential step toward understanding the physiological status of healthy paralarvae, knowledge, which can then be transferred to increase their survival in captive conditions.

## Materials and methods

This study was performed in accordance with existing Spanish guidelines and regulations on animal research (Ley 32/2007, November 7th), and was consequently exempt from an ethics review process.

### Sample collection

**Figure 1:**
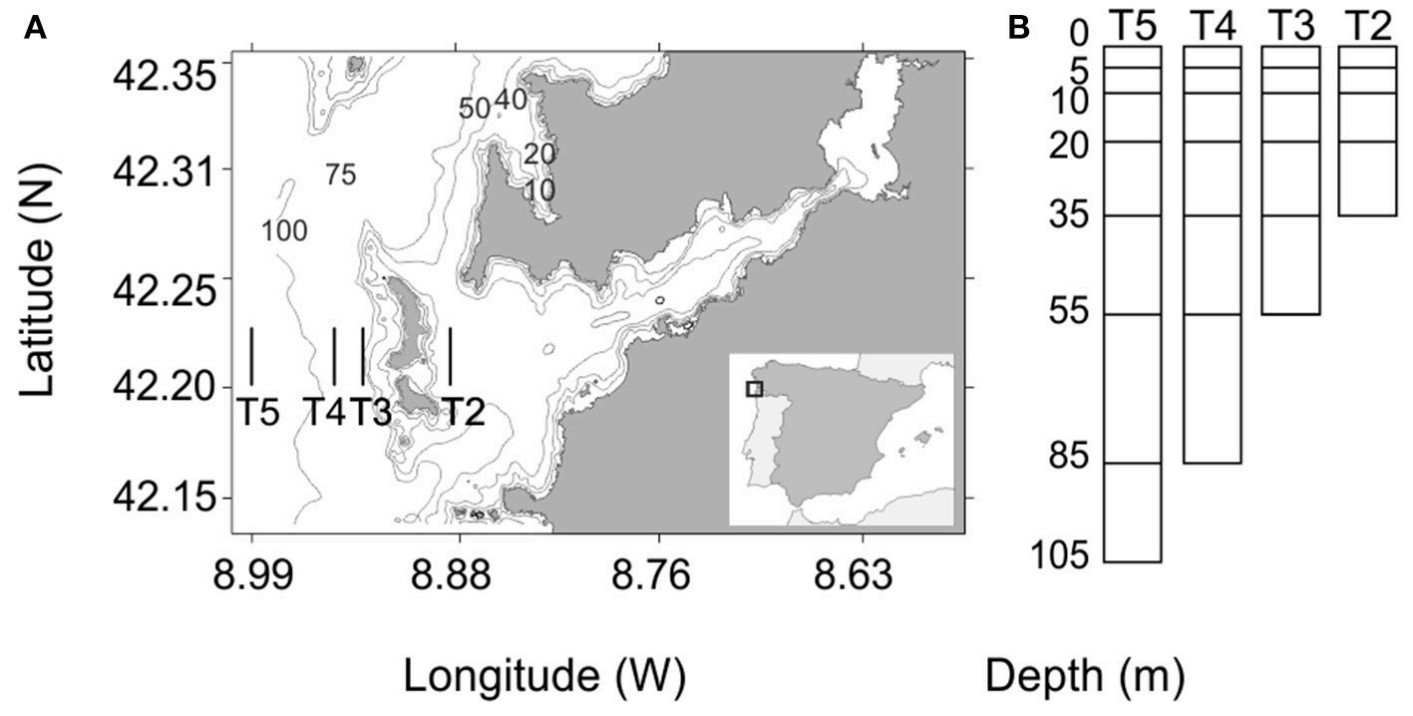
**(A)** Map of the study area showing the four transects performed in 2012 and 2014. **(B)** Depth layers sampled for each different transects.

Zooplankton samples were collected in the Ría de Vigo (NW Spain) onboard RV “*Mytilus*” in 2012 and 2014. The timing of the sampling was based on previously identified periods of maximum paralarval abundance (Rocha et al., 1999; González et al., 2005) in 2012 and 2014: we carried out ten nocturnal surveys each year, four in summer (July), and six in early autumn (September and October). Additionally, diurnal surveys were conducted in summer and in autumn 2012 (one per season). Sampling surveys were conducted along four transects (Figure 1A). For each transect, a Multinet® Hydrobios Mammoth of 250 μm mesh size, fitted with two electronic flow meters, was lowered at 2.5 knots to the sea floor and lifted up gradually to the surface. We defined seven depth layers: from 105 to 85 m, Z7; 85 to 55 m, Z6; 55 to 35 m, Z5; 35 to 20 m, Z4; 20 to 10 m, Z3; 10 to 5 m, Z2; and 5 m to the surface, Z1; see Figure 1B). Within each layer, the Multinet® filtered up to 200 m^3^ of seawater (approximately from 5–10 min for each layer and hence in total between 20 and 70 min), and collected independent samples. The collected zooplankton was fixed onboard in 96% ethanol and frozen at −20°C until sorting. In the laboratory, all cephalopod paralarvae were separated and preserved individually in 70% ethanol and stored at −20°C.

### Identification of paralarvae and morphological measurements

Dorsal mantle length (DML) was measured to the nearest 0.05 mm on the dorsal side of all octopus and squid paralarvae using a Leica M205C stereomicroscope and Leica Application System image analysis software (Leica Microsystems, Germany). All octopus paralarvae (*n* = 492) were identified as *O. vulgaris* based on morphological characters following Sweeney et al. (1992). Due to the difficulty of identifying squid paralarvae using morphological characters, all loliginid paralarvae (*n* = 163) were identified genetically. Molecular identification relies on previous work with adult specimens identified morphological and subsequently genetically.

Briefly, DNA from the mantle of each loliginid paralarva was extracted with a QIAamp DNA Micro Kit (QIAGEN) following manufacturer's instructions, with the exception of two steps: Digestion at 56°C was done overnight and the final elution was done in two steps using 15 μl buffer AE in each elution. The barcoding region of the Cytochrome c Oxidase subunit I (COI) was amplified with the universal primers HCO2198 and LCO1490 (Folmer et al., 1994) and PCR products were sequenced by Sanger sequencing (Stab Vida, Portugal). Each sequence was compared to the following GenBank reference sequences using the BLAST algorithm (Altschul, 2014): *Alloteuthis media*, EU668085 (Anderson et al., 2008); *A. subulata* EU668098 (Anderson et al., 2008), and *L. vulgaris*, KF369142 (Lobo et al., 2013).

Loliginid paralarvae were identified as *A. media* (*n* = 93), *A. subulata* (*n* = 35), and *L. vulgaris* (*n* = 22) (Olmos-Pérez et al., unpublished data). For dietary analysis, a total of 64 *O. vulgaris* paralarvae (summer, *n* = 26; autumn, *n* = 38) and 32 *A. media* (summer, *n* = 16; autumn, *n* = 16) were selected. *Alloteuthis media* was selected for molecular diet analyses because it was the most abundant loliginid present. Samples were chosen to maximize information from different seasons, transects and depth.

### Digestive gland dissection, DNA extraction, and prey detection

Digestive glands of all 96 paralarvae were dissected out, cleaned with sterile distilled water and placed into DNA-free tubes (Suzuki et al., 2006). DNA was extracted with a QIAamp DNA Micro Kit (QIAGEN, Hilden, Germany) following the modifications in the elution step as stated before. DNA purity and concentration were controlled with NanoDrop 2000c UV-Vis Spectrophotometer (Thermo Fisher Scientific Inc., Massachusetts, USA).

Many dietary studies recommend the use of restriction enzymes (Blankenship and Yayanos, 2005) or blocking primers (Vestheim and Jarman, 2008; Deagle et al., 2009; Leray et al., 2013a) to avoid amplifying predator DNA and maximize detection of prey DNA. However, the huge number of sequences currently obtained with NGS platforms allows the use of universal primers that facilitate the detection of unexpected prey (Boyer et al., 2013) without the necessity of using restriction enzymes or blocking probes (Piñol et al., 2014). Accordingly, we employed the universal pair of primers HCO2198 (Folmer et al., 1994) and mlCOIintF (Leray et al., 2013b), to amplify 315 base pairs (bp) of the barcoding region of the mitochondrial cytochrome c oxidase subunit I (mt-COI) gene (Table 1). Cycling conditions for the touch-down PCR with COI primers were: initial denaturation at 95°C 3 min, 10 initial cycles of denaturation at 95°C for 30 s, annealing for 30 s at 57 (−1°C per cycle), and extension at 72°C for 40 s, followed by 29 cycles of denaturation at 95°C for 30 s, annealing at 47°C for 30 s, extension at 72°C for 40 s, and a final elongation for 4 min at 72°C. PCR amplification was performed in a total volume of 25 μl:1 μl (10 μM) of each forward and reverse primers, 12.5 μl Thermo Scientific™ Phusion™ High-Fidelity PCR Master Mix with HF Buffer (Thermo Fisher Scientific Inc., Massachusetts, USA), 1 μl of DNA (20 ng/μl), and 9.5 μl H_2_O.

**Table 1:** **All of them amplify different regions of mitochondrial (mt) DNA. Product size (bp): Approximate product size of each PCR product (without overhang) expressed as number of base pairs (bp)**.

**Gen**	**Target**	**Forward**	**Reverse**	**Product size (bp)**	**References**
*mt-COI*	Universal	ml COIintF^a^	HCO2198^b^	315	Folmer et al., 1994^b^; Leray et al., 2013b^a^
		GGWACWGGWTGAACWGTWTAYCCYCC	TAAACTTCAGGGTGACCAAAAAATCA		
*mt-16Sa*	Malacostraca	16S1F^*^	16S2R^*^	205	Deagle et al., 2005
		TGACGATAAGACCCT	CGCTGTTATCCCTAAAGTAACT		
*mt-16Sb*	Chordata	Chord_16S_F_TagA	Chord_16 s_R_Short	155	Deagle et al., 2009
		ATGCGAGAAGACCCTRTGGAGCT	CCTNGGTCGCCCCAAC		

* *These primers include two nucleotide modifications from the original to amplify specifically the Malacostraca family. All primers were synthesized with the following overhang on the 5′ ends: Forward (5′–3′) TCGTCGGCAGCGTCAGATGTGTATAAGAGACAGTGACGATAAGACCCT and Reverse (5′–3′) GTCTCGTGGGCTCGGAGATGTGTATAAGAGACAGCGCTGTTATCCCTAAAGTAACT*.

Since decapods, krill and fishes had been previously detected in the digestive tract of *O. vulgaris* paralarvae (Passarella and Hopkins, 1991; Vecchione, 1991; Roura et al., 2012), we also employed two pairs of specific primers to amplify the 16S mitochondrial gene of malacostracan crustaceans (mt-16Sa, Table 1) and chordates (mt-16Sb; Table 1). Cycling conditions for both 16S pairs of primers were: initial denaturation at 94°C 15 min, 33 cycles of: denaturation at 94°C for 20 s, annealing at 48.7°C for 90 s, extension at 72°C for 45 s, and a final elongation step at 72°C for 2 min. PCR amplification was performed in a total volume of 25 μl:2 μl (10 μM) of each forward and reverse primers, 12.5 μl of Promega GoTaq® Green Master Mix (Promega Corporation, Wisconsin, USA), 1 μl of DNA (20 ng/μl) and 7.5 μl H_2_O.

All primers were synthesized following New Zealand Genomics Ltd. (NZGL) recommendations, with an overhang on the 5′ ends to permit the ligation with Illumina multiplexing indices and sequencing adapters (Table 1). The optimum annealing temperature with the overhangs was determined with a gradient PCR. For each primer, 2 μl of PCR product were checked on 1.5% agarose gels. Those that presented a clear band of expected size were cleaned up with Agencourt AMPure beads following the manufacturer's protocol (Beckman Coulter Life Science Inc., México). Afterwards, PCR products were quantified using a Qubit™ 3.0 fluorometer (Thermo Fisher Scientific Inc., Massachusetts, USA). Purified PCR products of the same individual with concentration higher than 1.0 μg/ml were pooled together. Library preparation with 96 Nextera Index Primers, quantification, normalization, and pooling were performed by New Zealand Genomics Ltd. (NZGL) in their laboratories. The library was then sequenced with MiSeq Reagent Kit V3 in MiSeq sequencer (Illumina Inc., USA).

### Bioinformatic analysis

MiSeq reporter was used to separate and remove the adapters for the 96 samples. The software Fastq-Multx (Aronesty, 2011) was used to demultiplex amplicons according to the primer sequences. Due to the wildcard characters in the primer sequences, up to 7 base pair mismatches were permitted. Software SolexaQA++ 3.1.4 was used to ensure the reads were still paired (Cox et al., 2010). The paired end reads (read 1 and read 2) were merged using VSEARCH 1.9.5 (Rognes et al., 2016). Paired reads that did not meet the following quality filtering were discarded (Edgar and Flyvbjerg, 2015): (i) reads with quality score over 3, (ii) reads longer than 140 bp, (iii) reads with less than one expected error in the primer sequence or barcodes. Unique sequences were clustered using a 97% identity threshold and remaining singleton Molecular Taxonomic Units (MOTUs) were discarded. Chimeric sequences were then removed using UCHIME (Edgar et al., 2011). Using the final list of representative sequences, each MOTU was searched against the GenBank database using BLAST 3.2.31 (Camacho et al., 2009).

MOTUs with BLAST query coverage under 60% or BLAST identities lower than 74% were also deleted from the database. Potential contamination and predator MOTUs (i.e., *A. media* and *O. vulgaris*) were removed from the database. Potential prey MOTUs were assigned using the following criteria to taxonomical categories: MOTUs with identity higher than 97% were determined at species level, MOTUs between 93 and 97% were assigned to genus, and MOTUs with identity below 93% were assigned to family.

### Statistical analysis

For each predator (*A. media* and *O. vulgaris*), we analyzed separately the MOTUs identified by different pairs of primers. Then, we calculated the proportion of reads for each MOTU in relation to the total number of reads (PR). We also calculated the frequency of occurrence for each MOTU (FM: percentage of number of samples tested positive for a given MOTU in relation to the total number of samples) and the frequency of the occurrence of each family (FF: percentage of number of samples tested positive for a given prey family in relation to the total number of samples).

Frequency of occurrence was calculated for higher taxonomic levels by combining information for all MOTUs falling within the relevant taxon (Table 2). Moreover, for those taxa that were detected with at least two pair of primers and to species level, we calculated the overall frequency of occurrence (percentage of samples which tested positive for a given taxon in relation to the total number of samples, Table 2).

**Table 2:** **Taxonomic groups identified with the different primers (COI, 16Sa or16Sb) in both predators**.

**Phyllum**	**Order**	**Family**	**Genus**	**Species**	**FT** ***O. vulgaris***				**FT** ***A. media***			
					**COI**	**16Sa**	**16Sb**	**O**	**COI**	**16Sa**	**16Sb**	**O**
Arthropoda	Amphipoda	Hyperiidae			9.38							
	Calanoida	Calanidae	*Calanoides*	*C. carinatus*	1.56							
		Candaciidae	*Candacia*	*C. armata*						6.25		
		Clausocalanidae	*Clausocalanus*	*C. jobei*	4.69				6.25			
			*Ctenocalanus*	*C. vanus*					6.25			
			*Pseudocalanus*	*P. elongatus*					9.38			
									6.25			
		Euchaetidae	*Paraeuchaeta*	*P. hebes*					3.13			
		Paracalanidae	*Paracalanus*	*P. parvus*					3.13			
					39.63				31.25			
	Decapoda	Alpheidae				1.56						
		Carcinidae	*Carcinus*	*C. maenas*	4.69	32.81		32.81		9.38		
						4.69						
						7.81						
		Crangonidae	*Crangon*	*C. crangon*		3.13						
		Diogenidae	*Diogenes*	*D. pugilator*	4.69							
						4.69						
		Galatheidae	*Galathea*	*G. intermedia*	3.13							
				*G. squamifera*		1.56						
					3.13							
		Goneplacidae	*Goneplax*	*G. rhomboides*	23.44	12.50		26.56	9.38	6.25		12.50
					7.81							
					9.38							
		Inachidae	*Macropodia*	*M. parva*		4.69						
			*Inachus*	*I. dorsettensis*		3.13						
						7.81						
					1.56	1.56						
		Paguridae	*Anapagurus*	*A. hyndmanni*	3.13							
			*Pagurus*	*P. bernhardus*	7.81	6.25		10.93		3.13		
				*P. prideaux*	3.13	1.56		4.68	6.25			
					1.56	3.13						
		Pilumnidae	*Pilumnus*	*P. hirtellus*	46.88	67.19		76.56	6.25	18.75		25.00
						15.63						
					12.50	6.25						
						4.69						
		Pirimelidae	*Pirimela*	*P. denticulata*		7.81						
						3.13						
		Polybiidae	*Liocarcinus*	*L. navigator*	3.13	6.25		6.25		3.13		
					6.25	4.69						
			*Necora*	*N. puber*	1.56	10.94		10.94	3.13	3.13		6.25
						1.56						
						4.69						
		Porcellanidae	*Pisidia*	*P. longicornis*	6.25	9.38		10.93	6.25	21.88		25.00
		Portunidae			14.06				3.13			
		Processidae	*Processa*	*P. nouveli holthuisi*	1.56							
				*P. edulis crassipes*		3.13						
		Sesarmidae			1.56							
		Thiidae	*Thia*	*T. scutellata*		1.56						
		Upogebiidae	*Upogebia*	*U. deltaura*	4.69							
						7.81				6.25		
		Varunidae				1.56				3.13		
		Xanthidae	*Xantho*	*X. pilipes*	1.56							
	Diplostraca	Podonidae	*Podon*	*P. intermedius*		9.38				6.25		
		Sididae	*Penilia*	*P. avirostris*	12.50							
						7.19						
	Euphausiacea	Euphausiidae	*Nyctiphanes*	*N. couchii*		4.69				18.75		
					1.56				12.50			
	Poecilostomatoida	Oncaeidae	*Oncaea*		4.69				3.13			
									6.25			
Chaetognatha	Phragmophora	Eukrohniidae			3.13				9.38			
Chordata	Perciformes	Gobiidae	*Crystallogobius*	*C. linearis*	1.56							
	Salpida	Salpidae	*Thalia*	*T. democratica*							3.13	
Echinodermata	Euryalida	Euryalidae			14.06				12.50			
	Ophiurida	Amphiuridae	*Amphiura*	*A. abyssorum*		7.81				3.13		
		Ophiactidae			4.69							
		Ophiuridae	*Ophiura*	*O. albida*						3.13		
					7.81				3.13			
									3.13			
Cnidaria	Leptothecata	Campanulariidae	*Obelia*	*O. geniculata*	1.56				46.88		12.50	
					1.56							
	Siphonophorae	Diphyidae	*Muggiaea*	*M. atlantica*	1.56				15.63			
			*Muggiaea*									
		Prayidae	*Rosacea*	*R. flaccida*			3.13				9.38	
Mollusca	Mytilida	Mytilidae	*Mytilus*	*M. galloprovincialis*		3.13						
	Architaenioglossa	Viviparidae			4.69							
	Veneroida	Lasaeidae			6.25							
		Montacutidae	*Tellimya*	*T. ferruginosa*	1.56							
Nermertina	Heteronemertea	Lineidae	*Cerebratulus*						3.13			

*The frequency of occurrence of each Taxon (FT), detected by the primers individually (COI, 16Sa, 16Sb) and the combined information for taxa detected by at least two pair of primers (O, Overall frequency of occurrence)*.

Redundancy Analysis (RDA) was used to detect patterns in the diet of *O. vulgaris* and *A. media* and determine which explanatory variables influenced those patterns. We included the occurrences of each family (FF) detected in the analysis as response variables, considering the presence-absence of each family in each paralarva. All octopus paralarvae presented three suckers per arm and were therefore probably less than 10 days old (Villanueva, 1995; Garrido et al., 2016b). All squid paralarvae were less than 41 days old based on statolith ring measurements (Olmos-Pérez et al., unpublished data). However, since we did not have complete age data, size-at-age is very dependent on environmental (pre- and post-hatching) temperature, and food ingestion is likely more dependent on size that on age, we categorized DML into three different classes to facilitate detection of ontogenetic changes during paralarvae growing. Thus, *O. vulgaris* paralarvae were categorized as small (1.20–1.74 μm; *n* = 21), medium (1.75–1.98 μm; *n* = 21), or large (1.99–2.28 μm; *n* = 22), and *A. media* were categorized as small (1.42–1.99 μm, *n* = 9), medium (2.00–2.99 μm; *n* = 15), or large (3.00–6.02 μm; *n* = 8). Paralarval size class (i.e., small, medium, large), transects (i.e., transects T2, T3, T4, T5), seasons (i.e., summer, early autumn), and depth (z1, z2, z3, z4, and z5) were included as nominal explanatory variables. We used the correlation triplot (α = 0, species conditional triplot), and the correlation matrix for the response variables. A significance test was applied with 4,999 permutations.

The effects of the season, transects, depth, and DML on dietary diversity and the presence of particular prey families in the diet were analyzed with generalized additive modeling (GAM) using a Poisson distribution for diversity and a binomial distribution for the other response variables and logit link function (link=logit), with season and transect as fixed factors and DML and depth effects fitted as smoothers (setting the bases dimension using *k* = 4 to avoid overfitting). Only the most frequently predated families (i.e., those detected in at least 10% of the predators, FF > 10) were used in this analysis. Models were fitted using backwards selection. The goodness-of-fit of the models was assessed with the Akaike Information Criterion (AIC). When the difference in AIC between two models (i.e., with and without one explanatory variable) was less than 2, an *F*-test was employed to select the best model (in case of a significant *F*-value the more complex model was preferred). All statistical analyses were performed with Brodgar 2.7.4. (Highland Statistics Ltd., UK).

Finally, “discovery curves” were plotted to determinate if the number of samples was sufficient to determine the importance of the most frequently detected prey species, for each combination of predator species (*O. vulgaris* and *A. media*), and primers (COI and 16sa). For each prey species, predator and primers, sets of 0, 1, 2… *n* samples were drawn at random from the available n samples and the proportional occurrence of the prey type was calculated for each sample size. Ten replicates were used to generate means and confidence limits, which were then plotted against sample size.

## Results

### Bioinformatic analysis

Of 8,274,658 raw reads, 5,734,163 were successfully demultiplexed and contained both read 1 and read 2. Then, 5,119,926 paired end reads were successfully merged, and 4,752,768 reads remained after quality filtering. A total of 4,124,464 reads was clustered into 1,155 MOTUs using a 97% threshold. Of the total reads, 3,189,247 corresponded to COI, 744,474 reads to the primers set 16Sa and 190,743 reads to the primer set 16Sb. Of these, 405 MOTUs (31,604 reads) did not match any GenBank sequence and 1,155 MOTUs, 750 (4,092,860 reads) had a match on GenBank database.

After meeting the thresholds of query coverage and identity, 465 MOTUs (131,843 sequences) were removed and 285 MOTUs (3,961,017 reads) were classified as: contamination (48 MOTUs, 41,422 reads), *O*. *vulgaris* (60 MOTUs, 2,150,967 reads), and *A. media* (55 MOTUs, 1,534,538 reads) and were removed prior to the analysis. In total, 122 MOTUs (234,090 reads) were then considered as potential prey.

Finally, 66 prey MOTUs (112,015 reads) were detected with the pair of primers COI, 53 prey MOTUs (122,029 reads) with pair of primers 16Sa and 3 prey MOTUs (46 reads) with pair of primers 16Sb (Supplementary Material 1). The total number of sequences in each category detected in both predators by different pair of primers was, the average and the range are presented in Supplementary Material 2.

### Prey identification

Genetic analyses with primer COI revealed the presence of prey in the digestive glands of 56 *O. vulgaris* (56/64 = 87.5%) and 25 *A. media* (25/32 = 78.1%). Primers 16Sa revealed the presence of prey in the digestive tracts of 51 *O. vulgaris* (79.7%) and 10 *A. media* paralarvae (31.3%). Finally, 16Sb revealed the presence of prey in 2 *O. vulgaris* (3.12%) and 7 *A. media* paralarvae (21.87%). Primers COI amplified a wide spectrum of species detected in gut content of cephalopods paralarvae, belonging to a minimum of 7 phyla, 15 orders, and 32 families. Within these higher taxa, we were able to distinguish 28 genera and 27 species (Table 2). Primers 16Sa amplified mainly decapods, but also species belonging to other taxonomic groups. In total, they amplified taxa belonging to a minimum of 3 phyla, 6 orders, and 22 families. Within these, we were able to distinguish 21 genera and 24 species (Table 2). Primers 16Sb amplified in total 2 phyla, 3 orders, 3 families. Within these, we were able to distinguish 3 genera and 3 species (Table 2). In total, 21 families were exclusively identified by COI primers, 9 by 16Sa primers, and 2 by 16Sb. Thirteen families were detected with both primers COI and 16Sa (Table 2).

In total, considering all pair of primers together, prey were detected in 62 (96.9%) *O. vulgaris* and in 26 (81.3%) *A. media* paralarvae. The number of different prey taxa identified in individual *O. vulgaris* paralarvae range between 0 and 9 (mean ± standard error, 2.1 ± 0.267) with COI and between 0 and 7 (2.22 ± 0.232) with 16Sa. In individual *A. media* the number of prey taxa identified with COI primers were between 0 and 8 (2.09 ± 0.334) and between 0 and 11 (0.94 ± 0.378) with 16Sa.

### O. vulgaris

**Figure 2:**
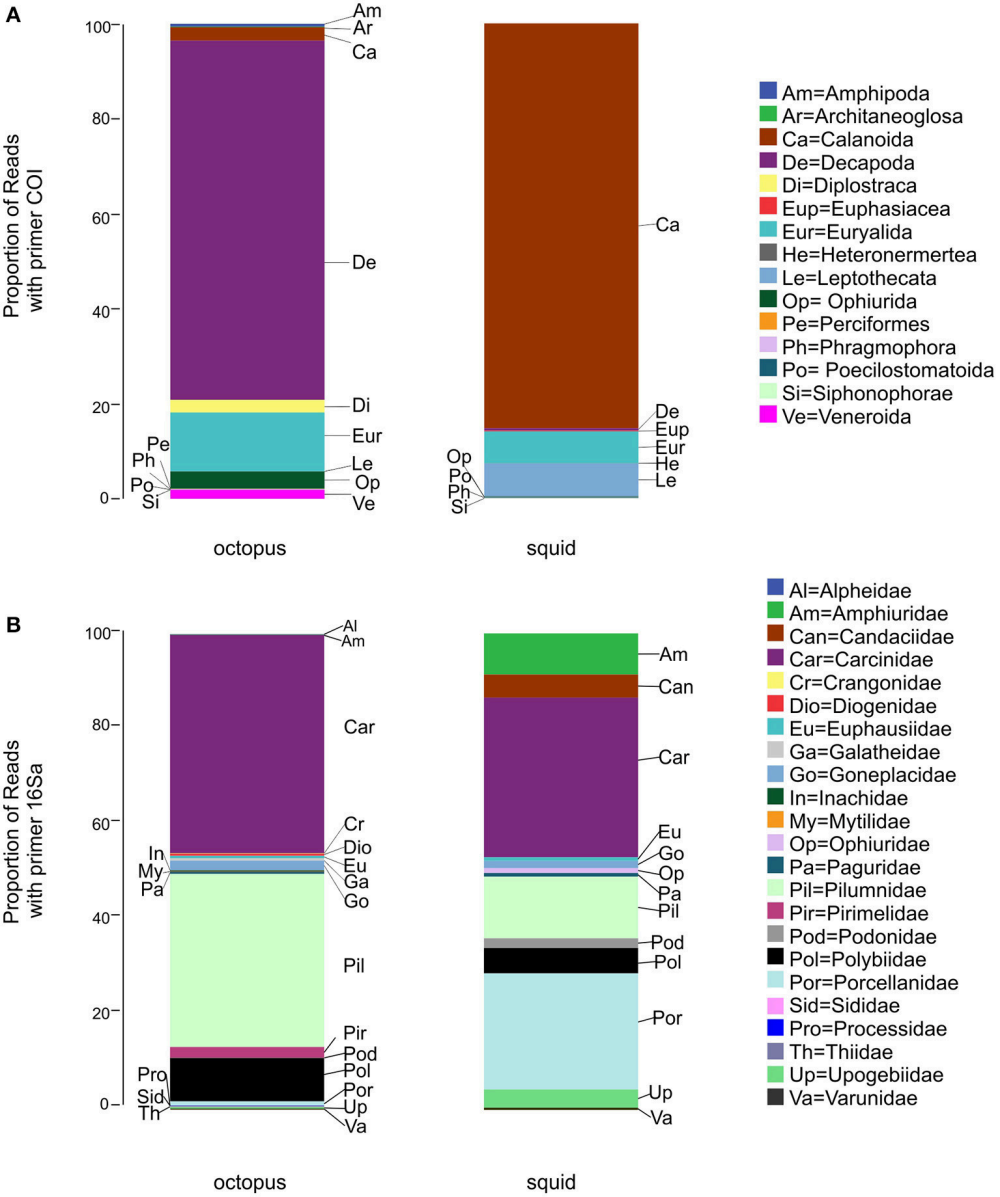
**Proportion of Molecular Operational Taxonomic Units (MOTU) prey reads (PR) detected with the primer COI (A)** and the primer 16Sa **(B)** in *O. vulgaris* and *A. media*. MOTUs were clustered in orders **(A)** or families **(B)**.

The most abundant prey reads detected with primers COI (Figure 2A) matched with the crabs *Goneplax rhomboides* (order Decapoda), an unknown species of the family Portunidae (order Decapoda) and *Pilumnus hirtellus* (order Decapoda), as well as an unknown ophiuroid of the family Euryalidae (order Euryalida). With 16Sa primers (Figure 2B) the most abundant prey reads matched with the crabs *Carcinus maenas* (family Carcinidae) and *P. hirtellus* (family Pilumnidae) (Supplementary Material 1).

COI primers identified a total of 54 unique MOTUs belonging to 6 phyla, 14 orders, and 31 families. Within these, we were able to distinguish 28 genus and 20 species while 16Sa primers identified a total of 47 MOTUs belonging to 3 phyla, 5 orders, and 20 families. Within these, we were able to distinguish 18 genus and 19 species (Table 2).

Using COI primers, the most frequently detected MOTUs (FM) in *O. vulgaris* were the crab *P. hirtellus* (family Pilumnidae), the copepod *Paracalanus* sp. (family Paracalanidae) and the crab *G. rhomboides* (family Goneplacidae). With 16Sa, the most frequently detected MOTUs (FM) were the decapods *C. maenas* and *P. hirtellus*. The remaining MOTUs were detected in less than 10 octopus paralarvae (Supplementary Material 1).

**Figure 3:**
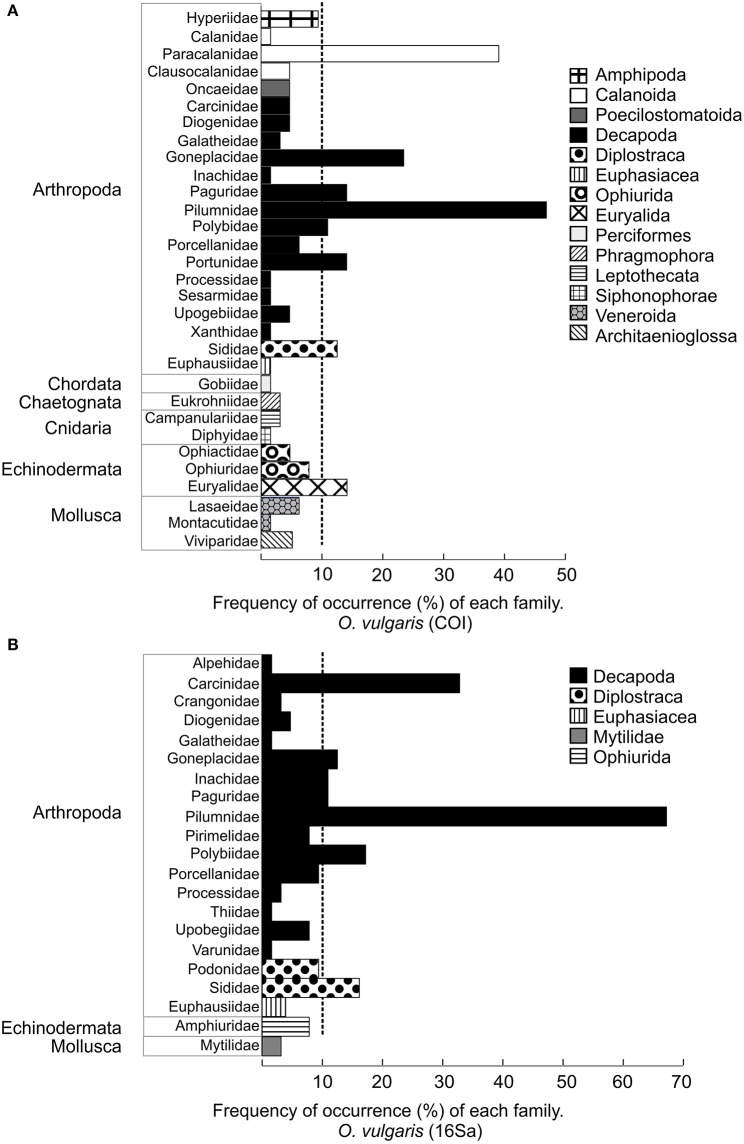
**Frequency of the occurrence of the families (FF) detected in ***O. vulgaris*** with primers COI (A)** and primers 16Sa **(B)** with primer COI. Colors represent different orders. The corresponding phylum is indicated on the left. Vertical dashed line indicates the families detected in more than 10% of paralarvae.

Pilumnidae was the most frequently detected family in *O. vulgaris* with primers COI and 16Sa (FF = 47 and 67%, respectively) (Figure 3). With primers COI, other families detected in more than 10% of *O. vulgaris* paralarvae were Paracalanidae, Goneplacidae, Paguridae, Portunidae, Euryalidae, Sididae, and Polybiidae (Figure 3A). Primers 16Sa detected the families Carcinidae, Goneplacidae, Inachidae, Paguridae, and Polybiidae in more than the 10% of octopus paralarvae (Figure 3B).

Analysis with the primer 16Sb revealed that *Rosacea flaccida* (Order Siphonophorae) was present in two *O. vulgaris* paralarvae (four reads).

### A. media

The most abundant prey reads with COI primers (Figure 2A) matched with the copepod *Paracalanus* sp. (order Calanoida), followed by the hydrozoan *Obelia geniculata* (order Leptothecata), and an unknown ophiuroid of the family Euryalidae (class Ophiuroidea). With 16Sa (Figure 2B), the most abundant prey reads matched with the crabs *C. maenas* (family Carcinidae), *Pisidia longicornis* (family Porcellanidae), and *P. hirtellus* (family Pilumnidae) (Supplementary Material 1).

In *A. media*, COI primers identified a total of 29 unique MOTUs, belonging to 5 phyla, 10 orders, and 17 families. Within these, we were able to distinguish 16 genera and 11 species, while 16Sa primers identified a total of 18 unique MOTUs, belonging to 2 phyla, 5 orders, and 13 families. Within these, we were able to distinguish 12 genera and 14 species (Supplementary Material 1).

The MOTUs most frequently detected (FM) in *A. media* were the hydroid *O. geniculata* (family Campanulariidae), the copepod *Paracalanus* sp. (family Paracalanidae), and the siphonophore *Muggiaea* sp. (family Diphyidae). The remaining 31 MOTUs were detected in less than 15% of squids (Table 2). 16Sa primers revealed that the most frequent detected MOTUs were *P. longicornis* and *P. hirtellus*. Seventeen MOTUs were detected in <10% of the squids (Supplementary Material 1).

**Figure 4:**
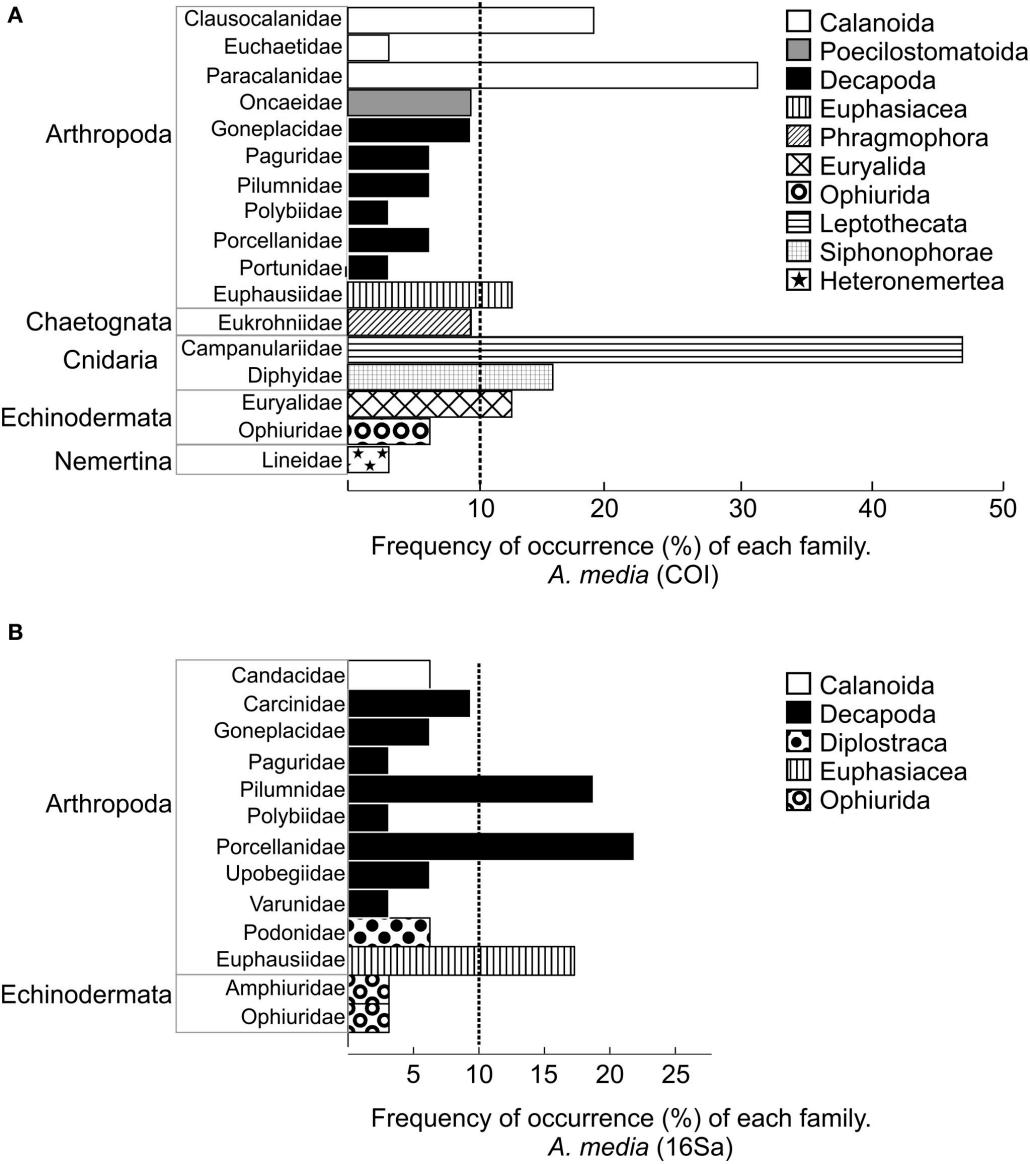
**Frequency of the occurrence of families (FF) detected in ***A. media*** with primers COI (A)** and with primer 16Sa **(B)**. Colors represent different orders. The corresponding phylum is indicated on the left. Vertical dashed line indicates the families detected in more than 10% of paralarvae.

The most frequently detected families detected with COI primers were: Campanulariidae (order Leptothecata), Paracalanidae, Clausocalanidae (order Calanoida), Diphyidae, Euphausiidae, and Euryalidae (Figure 4A). The most frequently detected families detected with 16Sa primers were Pilumnidae and Carcinidae (Figure 4B).

In *A. media*, 16Sb revealed the presence of the hydrozoan *O. geniculata* (FM = 12.5%, 32 reads), the siphonophore *R. flaccida* (FM = 9.3%, 7 reads), and the salp *Thalia democratica* (FM = 3%, three reads) (Supplementary Material 1).

### Diet selection

**Figure 5:**
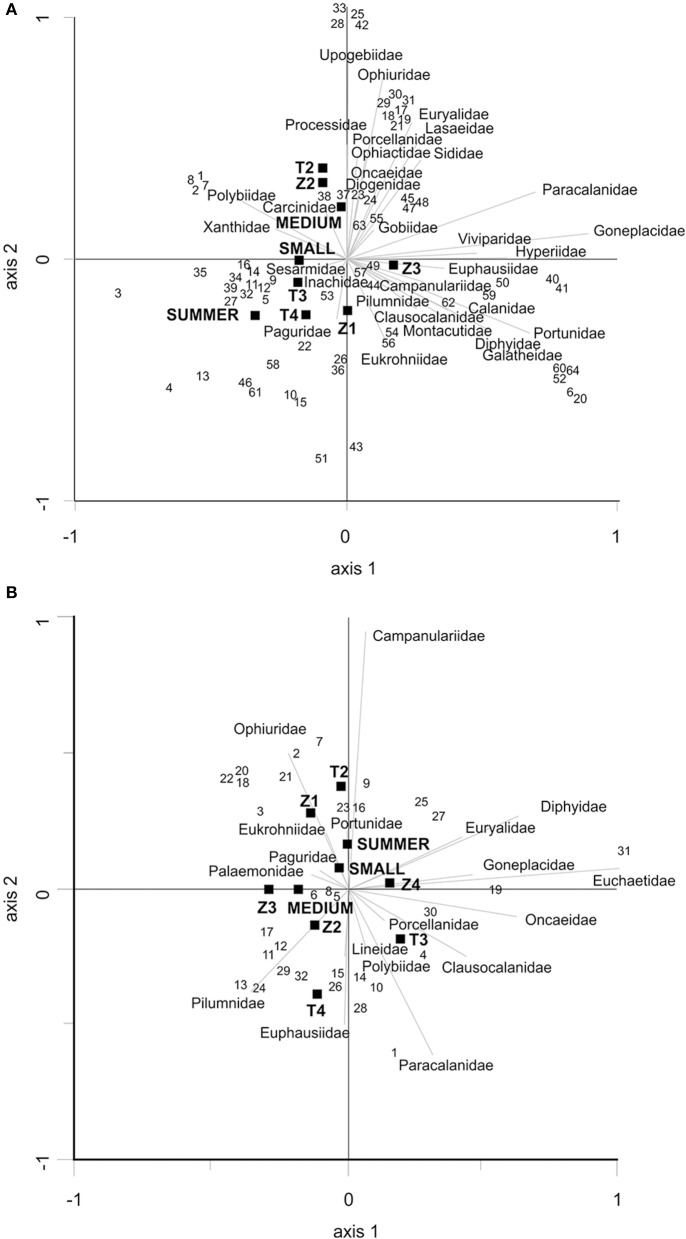
**(A)** RDA triplot for the *O. vulgaris* families identified with COI gene. The correlation matrix was used. The first axis explains 29.57% and the second axis explains 21.96% of the total sum of all canonical eigenvalues (0.192). All the explanatory variables were used. **(B)** RDA triplot for the *A. media* families identified with COI gene. The correlation matrix was used. The first axis explains 27.26% and the second axis explains 21.47% of the total sum of all canonical eigenvalues (0.39). All the explanatory variables were used.

RDA analysis showed that season and transect, but not depth or individual size, significantly affected the prey families detected in the diet of *O. vulgaris* (Table 3). The sum of all canonical eigenvalues was 0.192 and the first two axes accounted for 51.53% of the fitted variation (i.e., the 9.91% of the total variation in the family data; Figure 5). In *A. media*, transect, but not season, size, or depth, significantly affected the families detected in their diet (Table 3). The sum of all canonical eigenvalues was 0.309, and the first two axes accounted for 48.73% of the fitted variation (i.e., the 15.05% of the variation in the family data; Figure 5).

**Table 3:** **Summary of the RDA analysis**.

**Predator**	**Explanatory variable**	**Eigenvalue**	***F*-statistic**	***p*-value**
*O. vulgaris*	Summer	21.17	2.632	<**0.001**
	T2	14.39	1.824	**0.002**
	T3	9.76	1.610	**0.032**
	T4	7.35	1.009	0.447
	Z1	10.64	1.020	0.428
	Z2	13.43	1.437	0.060
	Z3	8.87	0.744	0.617
	Small	9.69	1.101	0.323
	Medium	9.28	1.289	0.109
*A. media*	Summer	11.51	1.556	0.080
	T2	18.57	1.826	**0.017**
	T3	13.27	0.379	0.970
	T4	10.80	0.900	0.548
	Z1	1.78	0.569	0.724
	Z2	4.73	0.836	0.529
	Z3	8.55	0.396	0.977
	Z4	14.41	0.522	0.079
	Small	8.39	1.079	0.368
	Medium	8.39	0.893	0.593

*Eigenvalues for single explanatory variables are expressed as a % of the sum of eigenvalues for the full set of explanatory variables (0.192 in O. vulgaris and 0.309 in A. media). All explanatory variables were included in the analysis: Season (Summer- Autumn); Transect (T2-T3-T4-T5); Depth (z1: 0-5 m; z2:5-10 m; z3: 10-20 m; z4: 20-35 m; z5:35-55 m); Size (small, medium, large). Significant values are in bold*.

**Figure 6:**
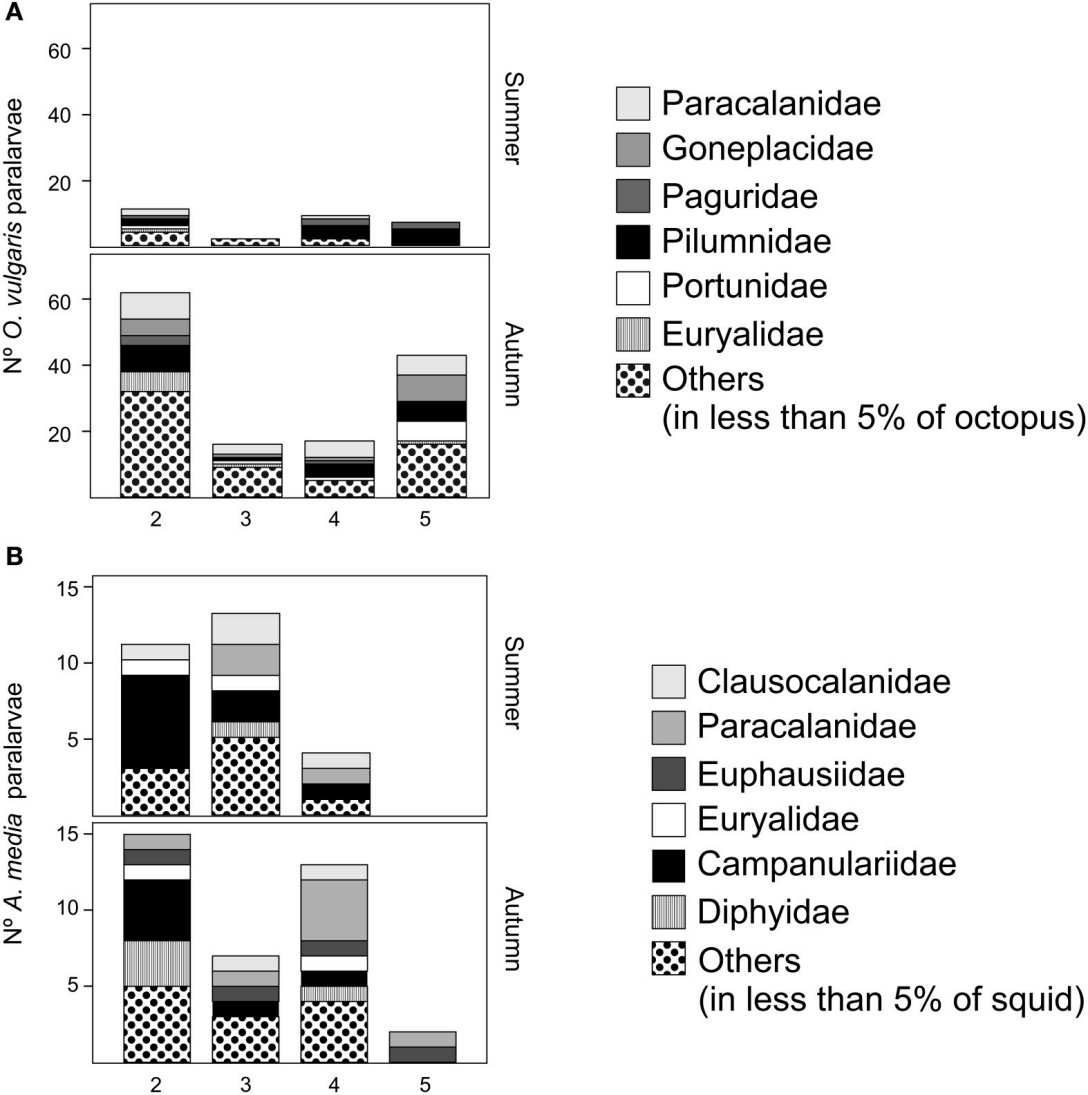
**Families detected in ***O. vulgaris*** paralarvae (A)** and ***A. media*** paralarvae **(B)** in different transects (2, 3 4, 5) and different seasons (summer, autumn). The vertical axis represents the number of paralarvae that present a given family.

**Figure 7:**
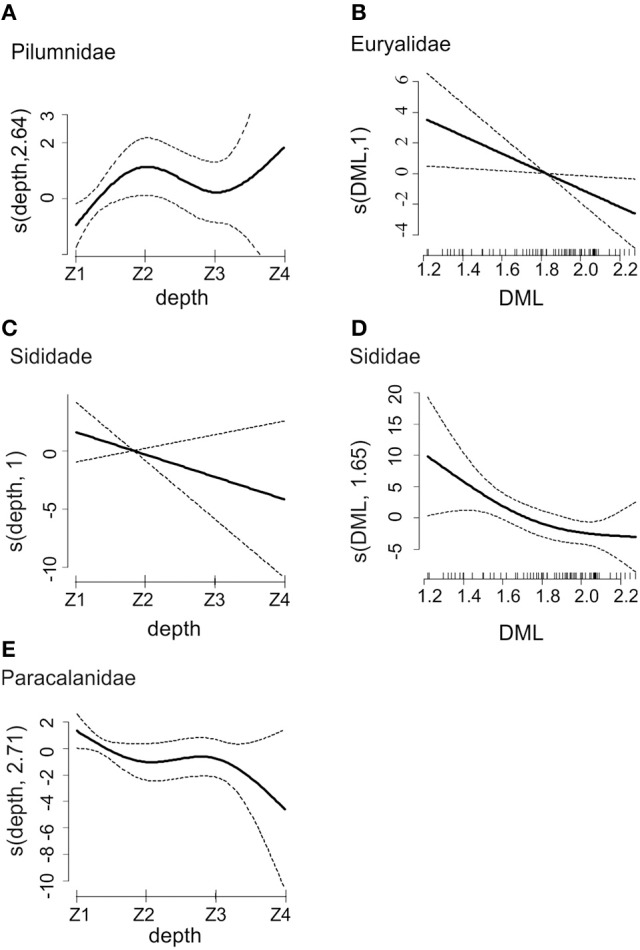
**Smooth curves for partial effects obtained by the Generalized Additive Modeling (GAM) of the occurrence of the families Pilumnidae (A)**, Euryalidae **(B)**, Sididae **(C,D)**, and Paracalanidae **(E)** as prey of *O. vulgaris* paralarvae. Explanatory variables used were dorsal mantle length (DML) and depth (Z1, Z2, Z3, Z4). Dotted lines are 95% confidence bands. The vertical axis represents the effect on the response variable.

The GAM analysis for the most frequently occurring families (detected in at least 10% of paralarvae) in *O. vulgaris* revealed that the copepod family Paracalanidae (FF = 39%) was more frequently predated in autumn than in summer (*p* < 0.001; Table 4). Predation on the crab family Pilumnidae (FF = 47%) differed across transects (*p* = 0.028; Table 4), being more frequent in T5 and T4 than the rest of transects (Figure 6). The ophiuroid family Euryalidae (FF = 14%) was more frequently predated in autumn than in summer (*p* = 0.010, Table 4) and was more frequent in smaller individuals (*p* = 0.020; Figure 7). The cladoceran family Sididae (FF = 13%) was more frequently found in small individuals (*p* = 0.030, Figure 7) and only detected in autumn (Table 4). The decapod families Goneplacidae (FF = 23%), Portunidae (FF = 14%), Paguridae (FF = 14%), and Polybiidae (FF = 11%) did not differ between seasons, among sizes or transects (*p* > 0.05 in all cases). The number of families in the diet of *O. vulgaris* differed between seasons (*p* < 0.001; Table 3) and among transects, (*p* = 0.023; Table 3). Thus, a wider range of families was predated in autumn than in summer, and in T3 than in T2 or T5 (Table 3). DML or depth did not affect the number of prey families detected in *O. vulgaris* (*p* > 0.05 in both cases).

**Table 4:** **Summary of the best GAMs fitted to (A) dietary diversity (number of taxa detected) and (B) the occurrence of different families detected in ***Octopus vulgaris*****.

		**Prey category**	**Model variables**		**Estimate**	**SE**	**Test**				***p*-value**	**Dev.exp**.	**AIC**
			**Compared level**	**Reference level**			**z**	**χ^2^**	**edf**	**df**			
**(A)**													
		Number of families	Season					34.460		1	<**0.01**	46.500	227.510
			Summer	Autumn	1.207	0.206	5.870				<**0.01**		
			Transect					9.570		3	**0.023**		
			T3	T2	−0.753	0.264	2.857				**0.004**		
			T4	T2	−0.291	0.229	−1.270				0.204		
			T5	T2	−0.013	0.184	−0.073				0.942		
			T4	T3	0.462	0.308	1.500				0.134		
			T5	T3	0.740	0.275	2.689				**0.007**		
			T5	T4	0.243	0.243	1.142				0.253		
**(B)**													
Phylum Arthropoda	Order Calanoida	Paracalanidae	s(depth)					5.050	2.711		0.162	33.700	72.180
			Transect					5.985		3	0.112		
			Season					11.440		1	**0.001**		
			Summer	Autumn	2.350	0.696	3.383				**0.001**		
	Order Decapoda	Pilumnidae	s(depth)					7.287	2.641		0.059	24.900	79.730
			Transect					9.081		3	**0.028**		
			T3	T2	−1.276	1.195	−1.068				0.286		
			T4	T2	1.673	0.896	1.867				0.062		
			T5	T2	1.837	0.845	2.173				**0.030**		
			T4	T3	2.949	1.282	2.301				**0.021**		
			T5	T3	3.113	1.255	2.481				**0.013**		
			T5	T4	0.163	0.850	0.193				0.847		
	Order Diplostraca	Sididae	Season					0.000		1	1.000	59.400	34.880
			Transect					0.000		1	1.000		
			s(DML)					7.097	1.648		**0.030**		
			s(depth)					1.544	1.000		0.214		
Phylum Echinodermata	Order Euryalida	Euryalidae	Season					6.595		1	**0.010**	23.000	46.010
			Summer	Autumn	4.303	1.676	2.568				**0.010**		
			s(DML)					5.380	1.000		**0.020**		

*Taxa listed are those for which the best GAM included a significant effect of at least one explanatory variable. Tests of significance are reported (χ^2^ or z-values and associated probabilities) for all explanatory variables retained in the final models. Degrees of freedom (df) are presented for parametric terms (season and transect) and estimated degrees of freedom (edf) for smoothers (dorsal mantle length (DML) and depth). An edf of 1 indicates a straight line relationship. In addition, parameter estimates, ±standard error (SE), z-statistic and p-values are presented to allow the comparison between the different levels of the nominal explanatory variables (season and transects). Significant values are in bold. Percentage of deviance explained (Dev. exp.) by the model and AIC are also presented*.

The GAM analysis of the most frequent families (detected in at least 10% of paralarvae) in *A. media* revealed that the occurrences of the families Campanulariidae (FF = 47%), Paracalanidae (FF = 31%), Clausocalanidae (FF = 19%), Diphyidae (FF = 16%), and Euryalidae, (FF = 13%) in the diet were not affected by size, depth, transect, or season (*p* > 0.005 in all cases). However, family Euphausiidae (FF = 13%) was only detected in autumn. The number of families in the diet did not differ significantly with paralarval size, depth, transect, or season (*p* > 0.05 in all variables).

**Figure 8:**
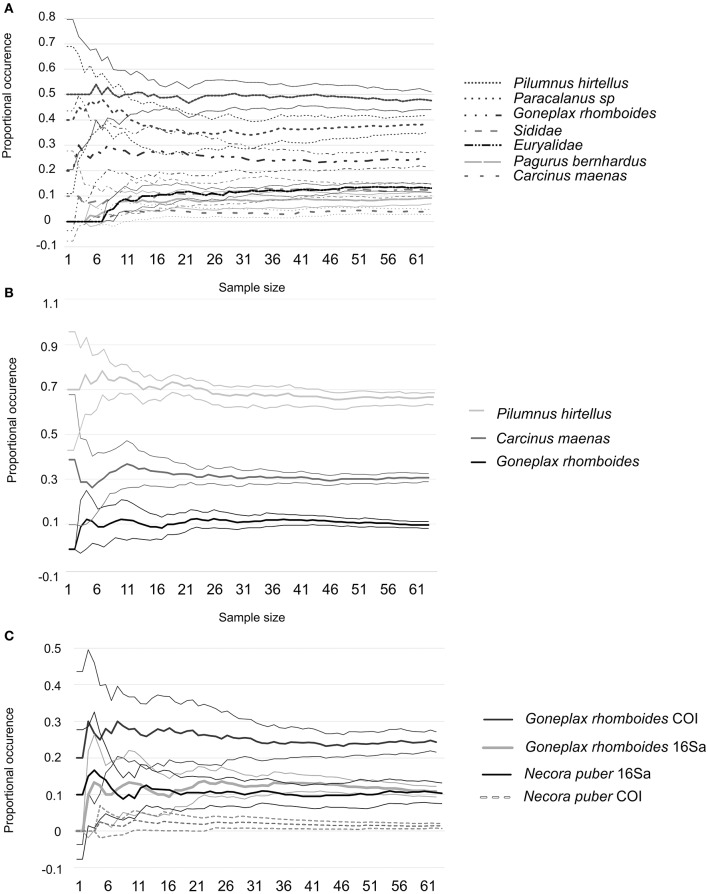
**Summary from 10 randomized sample sets of species identified in ***O. vulgaris*** with primers COI (A)**, primers 16Sa **(B)**, and both pair of primers **(C)**. Average values are presented as continues lines and Confidence Intervals (C.I.) as dashed lines.

**Figure 9:**
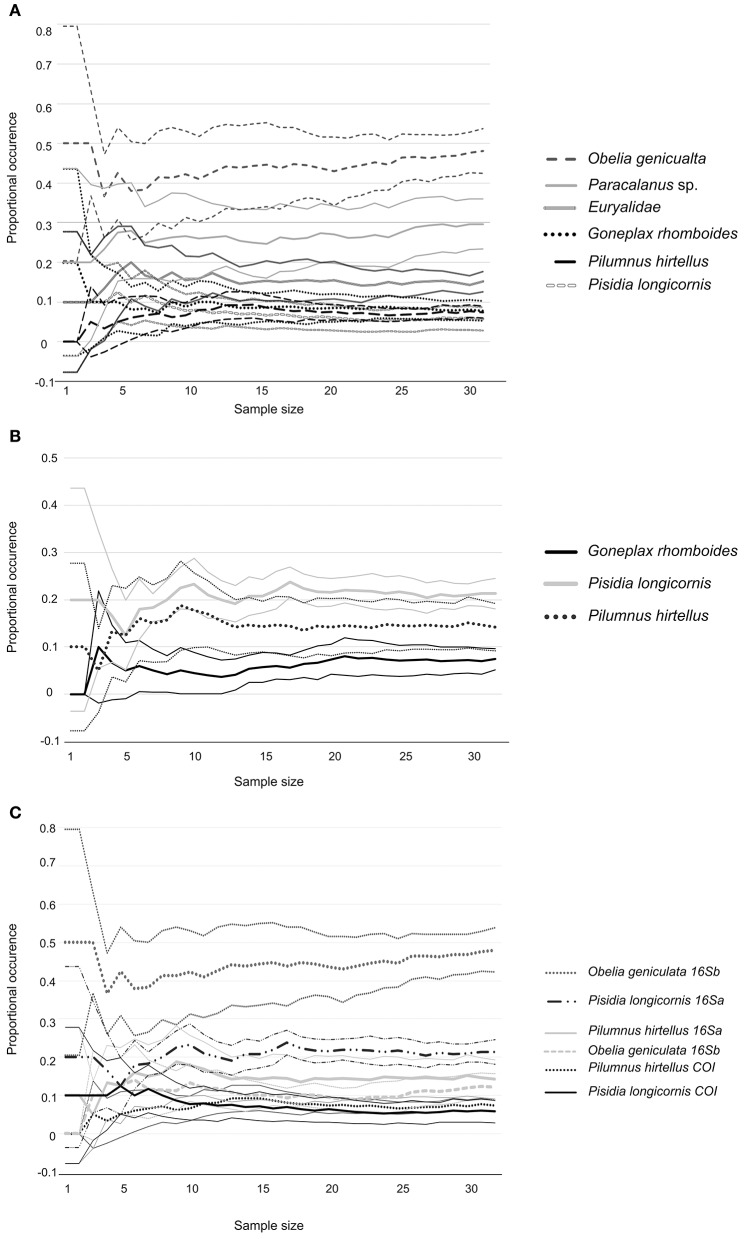
**Summary from 10 randomized sample sets of species identified in ***A. media*** with primers COI (A)**, primers 16Sa **(B)**, and both pair of primers **(C)**. Average values are presented as continues lines and Confidence Intervals (C.I.) as dashed lines.

The discovery curves for *O. vulgaris* (Figure 8) showed stabilization of the proportional occurrence estimates, when at least 45 of 64 paralarvae were sampled. The discovery curves for *A. media* (Figure 9), did not show any stabilization for the whole number of samples analyzed (*n* = 32).

## Discussion

Overall, 107 MOTUs were successfully identified in *O. vulgaris*, which corresponded to 40 different families, 31 genera, and 32 species, while in *A. media*, 58 MOTUs were identified corresponding to 25 different families, 23 genera, and 21 species (Supplementary Material 1). The combination of the different primers targeting small DNA fragments, and comprehensive genetic databases, permitted us to identify up to 77 types of prey (Table 2). For the first time, a molecular approach was successfully applied to identify prey of wild *A. media* paralarvae, thereby increasing the range of known prey of wild *O. vulgaris* paralarvae during their first days of planktonic stage. Together, the results increased the knowledge of the prey predated by cephalopod paralarvae in their natural environment, suggesting more species to feed paralarvae in captivity conditions.

The amplification of the COI barcoding mitochondrial region with universal primers detected a broader taxonomic range of prey than the 16S primers (Table 2), and allowed the identification of 21 families that were not amplified with 16S primers. Additionally, 16Sa primers detected prey in digestive glands where no prey was detected with COI primers. 16Sa primers also amplified nine additional families not detected with COI primers. Of those, four families belonged to the class Malacostraca which was their target (Deagle et al., 2005), but 16Sa primers also amplified cephalopod DNA and five prey families belonging to ophiuroids, copepods, cladocerans, and mollusks (Amphiuridae, Ophiuridae, Candaciidae, Podonidae, and Mytilidae, respectively). Lastly, primers 16Sb were specifically designed to amplify teleost fishes (Deagle et al., 2009) but they amplified DNA from the predator species (i.e., *O. vulgaris* or *A. media*), two cnidarian species and urochordates (i.e., salps). When the same taxa were detected by two primer pairs, they were usually amplified unequally in the same predator (i.e., different occurrence for each prey in the same paralarvae and different number of reads). These results could be explained due to low prey DNA quantity and differential affinity of primers to prey DNA, supporting the usefulness of including more specific primers to increase taxonomic resolution of prey ingested (Blankenship and Yayanos, 2005; Deagle et al., 2009).

Previous studies (Piñol et al., 2014) showed that blocking primers are not essential in molecular dietary studies to detect small quantities of prey DNA. In our study, despite the large quantity of predator sequences (90% of sequences), the 7.5% of reads obtained from potential prey (Supplementary Material 2), provided prey information never uncovered by other methods employed to the date (i.e., visual, cloning, immunoassay) and highly increased our knowledge about the diet of paralarvae with many new prey taxa recorded. The addition of blocking primers, could have diminished predator sequences, increasing the number of prey reads (Vestheim and Jarman, 2008; Deagle et al., 2009; Leray et al., 2013a) and might have revealed additional prey species. However, additional studies comparing prey identification in diet analysis with both methodologies would be necessary to assess the utility of blocking primers to analyze the diet of cephalopod paralarvae. Owed to the high sensitivity of NGS methodologies, it is important to underline the possibility of detecting DNA of other organisms that were consumed by the prey ingested by the paralarvae, i.e., secondary predation (Sheppard et al., 2005). In addition, it may happen that some of the prey detected could be captured by the paralarvae inside of the net. If so, it should be expected to find prey remains in the proximal part of the digestive tract (esophagus, stomach, or crop). However, since only the digestive gland was dissected, we can assume that the prey detected in this study was ingested by the paralarvae before their capture.

### Dietary differences

*Octopus vulgaris* paralarvae mainly preyed on decapod species, that generally comprise <5% of the total zooplankton abundance in the Ría de Vigo (Roura et al., 2013; Buttay et al., 2015). Among decapods, the species most frequently detected in *O. vulgaris* were the crabs *C. maenas, P. hirtellus*, and *G. rhomboides* (families Carcinidae, Pilumnidae, and Goneplacidae, respectively), that are also the most abundant decapod species in the Iberian Peninsula coast (Paula, 1987; Fusté and Gili, 1991; Queiroga, 1996). Family Pilumnidae was less frequent in more oceanic transects (T5), probably because they migrate from estuarine zones to offshore waters during their larval development and there is higher concentration in more inshore waters. Moreover, species of this family were more frequently detected in paralarvae captured at depths between 5 and 10 m, probably because they migrate to the upper water layers at night (Dos Santos et al., 2008).

The second most frequently detected group in *O. vulgaris* gut contents were the Calanoid copepods, a group not detected in previous studies (Roura et al., 2012). In particular, *Paracalanus* sp. was the main copepod identified in *O. vulgaris* gut. In Galician zooplankton communities, Calanoid copepods in general represent more than 60% of total zooplankton abundance (Blanco-Bercial et al., 2006; Roura et al., 2013; Buttay et al., 2015). Zooplankton community studies in this area have also shown that high abundances of *Paracalanus* species are linked to low salinity values (Blanco-Bercial et al., 2006). In our study, this prey was more frequently detected in *O vulgaris* paralarvae captured in autumn. The upwelling conditions during this season (i.e., cold and low salinity waters), could have promoted high abundances of this species increasing their availability in the environment and thus facilitating the predation.

Brittle stars (family Euryalidae) and cladocerans (family Sididae) were both frequently detected in small *O. vulgaris* paralarvae, perhaps because they are an easier target than fast moving copepods and decapods. The cladoceran identified with COI primers was *Penilia avirostris*. This species has been highlighted as an indicator of warm waters, and high abundances have occasionally been described in the Ría de Vigo associated with an increase in water temperature (Figueiras et al., 2011). The sea surface warming trend observed in Galician coastal waters during recent years (Gómez-Gesteira et al., 2008) could be favoring the presence of this cladoceran species. Another cladoceran that is very abundant in the Ría de Vigo was detected by primers 16Sa, namely *Podon intermedius* (Roura et al., 2013; Buttay et al., 2015). It was identified also in small and medium individuals. The detection of abundant cladoceran species in octopus guts could suggest opportunistic predation on cladocerans, specifically by smaller paralarvae.

Only one fish species was identified with COI primers in a single *O. vulgaris* paralarvae, and no fish DNA was amplified with 16Sb primers that were specifically designed to amplify fish DNA (Deagle et al., 2009). This result suggests low predation on fish, perhaps because the high mobility of fish larvae makes it difficult for the paralarvae to capture them.

Regarding squids, in *A. media* different prey species and different frequencies of occurrence were detected compared to *O. vulgaris*: Cnidarians were detected in *A. media* paralarvae of all sizes. Cnidarians are not very abundant in zooplankton community in Galicia (Buttay et al., 2015). Thus, these results could suggest selective predation on cnidarians, as also observed in turtles and sunfish (Dodge et al., 2011; Sousa et al., 2016). In contrast, cnidarians were only detected in three *O. vulgaris*. Their rare presence might be explained as a secondary predation effect (Sheppard et al., 2005) because high resolution of NGS, can detect small DNA amount present in the digestive tract of a prey captured by the paralarvae. It is also possible that hydroids are predated by *O. vulgaris* because they are easy to capture for slow recently hatched paralarvae (<10 days old, Garrido et al., 2016b). Moreover, squid paralarvae ingested up to ten copepod species, while only four were detected in octopus. This difference between *A. media* and *O. vulgaris* might be related with their hunting skills, which are developed during initial life stages (Villanueva et al., 1997). *Alloteuthis media* also preyed on decapods, and species of this group were mainly detected with the primer pair 16Sa. Thus, this could imply that the amount of DNA present was low and it was only possible to amplify decapod DNA with the specific pair of primers.

Other prey detected in both cephalopod species such as amphipods, cladocerans, euphausiids, and fishes, had been previously detected in the paralarval digestive system, but with a lower taxonomical resolution (Passarella and Hopkins, 1991; Vecchione, 1991; Venter et al., 1999; Vidal and Haimovici, 1999; Roura et al., 2012). Additionally, the gut contents of paralarvae of both species included molluscs, echinoderms, chaetognaths, and a nemertean that had never been previously identified in cephalopod paralarvae. Finally, DNA of chaetognaths and nemerteans was detected in a small number of paralarvae gut contents, and thus could reflect opportunist predation or alternatively, their DNA might be present in an organism ingested by the paralarvae, as an effect of secondary predation as explained above.

Diet diversity for *O. vulgaris* was influenced by the season and distance to shore. Numerous studies have shown that zooplankton communities in Galicia change according to oceanographic and meteorological conditions (Bode et al., 2009; Roura et al., 2013; Buttay et al., 2015). Thus, diet variability observed in *O. vulgaris* paralarvae might be related to zooplankton changes in prey availability in the zooplankton community. In contrast, no relationship could be established between the diet of *A. media* and the environmental explanatory variables or individual size. This may be related to small number of samples analyzed: discovery curves in *A. media*, showed very wide C.I. and no stabilization of the proportional occurrence estimates for the whole number of samples analyzed (*n* = 32). In contrast, *O. vulgaris* discovery curves, showed narrower C.I. and a stabilization of the proportional occurrence estimates, when at least 45 paralarvae are sampled. These results suggest that the number of *A. media* paralarvae analyzed was insufficient for a comprehensive dietary analysis of this species. In contrast, results suggest that the number of paralarvae of *O. vulgaris* analyzed in this study could be enough for this dietary analysis.

Our results showed that *O. vulgaris* prey on a wide variety of decapod species, but also frequently prey on other taxonomic groups, including mollusks, ophiuroids, amphipods, cladocerans, copepods, chaetognaths, or cnidarians. However, the low number of samples analyzed in previous research could have prevented the identification of rarely detected prey, that would likely only be identified when increasing the number of paralarvae analyzed. Moreover, the employment of several primers targeting different genes, could have favored the detection of additional species with broader taxonomic range that previous studies.

Overall, our results showed the usefulness of the NGS approach with several primers targeting different genes to dietary analysis of wild cephalopod paralarvae. Results have shown that they feed on a wide diversity of prey, mainly decapods, copepods, and cladocerans, but also other taxa that have not been previously identified in wild cephalopod paralarvae such as mollusks, echinoderms, chaetognaths, salps, cnidarians, and a nemertean. This study provides essential data to elaborate more suitable diets for captive cephalopod paralarvae, with the aim of increasing their survival for economically sustainable farming. Further studies are needed, including use of a wider variety of prey, mainly copepods from the genus *Paracalanus*, Cladocerans, and different decapod species, to test the effect on the digestive gland performance, growth and survival of recently hatched paralarvae.

## Author contributions

ÁG: conceived the plankton sampling strategy and financially supported the project; ÁG, ÁR, and LO-P: undertook sampling surveys and contributed to the conception of the experiment; SB, GP, and LO-P: planned the experimental design; LO-P and SB: executed the laboratory work; SB: handled bioinformatic data analysis; GP and LO-P: did statistical analysis. All the authors have revised the manuscript critically for important intellectual content and have approval the final version to be published.

## Funding

This study was supported by the project LARECO (CTM2011-25929) and CALECO (CTM2015-69519-R) funded by the Spanish Ministry of Economy and Competitiveness. LO-P was supported with a FPI grant (BES – 2012-055651) and a mobility grant (EEBB-I-15-10157) funded by the Spanish Ministry of Economy and Competitiveness. ÁR was funded with a postdoctoral grant from the “Fundación Barrié” and with RFWE funds from La Trobe University (Australia). We acknowledge support of the publication fee by the CSIC Open Access Publication Support Initiative through its Unit of Information Resources for Research (URICI).

### Conflict of interest statement

The authors declare that the research was conducted in the absence of any commercial or financial relationships that could be construed as a potential conflict of interest.
