# Supplementary material for: Diet Composition and Variability of Wild Octopus vulgaris and Alloteuthis media (Cephalopoda) Paralarvae: a Metagenomic Approach
Source: Front Physiol. 2017 May 24;8:321. doi: 10.3389/fphys.2017.00321 (PMC5442249; doi:10.3389/fphys.2017.00321)
Supplement: Supplementary file 1 [file Table1.DOCX]

**Supplementary material 1**

Taxa identified in *O. vulgaris* and *A. media* with pair of primers COI and 16Sa and 16Sb. All taxa are classified at phylum, order and family level. When possible, each taxon was classified to genus and species level. For each taxon, the corresponding number of MOTUs (MOTUs), the percentage of similarity with a GeneBank sequence (Id), percentage that overlaps with a GenBank sequence (QC), number of reads identified (Reads) and number of paralarvae in which the taxon has been identified (Occ.) are indicated. A total of 64 *O. vulgaris* paralarvae and 32 *A. media* paralarvae were analyzed

| **Phylum** | **Order** | **Family** | **Genus** | **Species** | | **Reads** | | **Occ** | **MOTUs** | **Id** | **QC** |
| --- | --- | --- | --- | --- | --- | --- | --- | --- | --- | --- | --- |
| ***O. vulgaris* COI gene** | | | | | | | | | | | |
| Arthropoda | Amphipoda | Hyperiidae |  |  | | 469 | | 6 | 1 | 93 | 81 |
|  | Calanoida | Calanidae | *Calanoides* | *Calanoides carinatus* | | 31 | | 1 | 1 | 99 | 93 |
|  |  | Clausocalanidae | *Clausocalanus* | *Clausocalanus jobei* | | 94 | | 3 | 2 | 99 | 90 |
|  |  | Paracalanidae | *Paracalanus* |  | | 1988 | | 25 | 3 | 96 | 92 |
|  | Decapoda | Carcinidae | *Carcinus* | *Carcinus maenas* | | 108 | | 3 | 1 | 100 | 92 |
|  |  | Diogenidae | *Diogenes* | *Diogenes pugilator* | | 147 | | 3 | 1 | 100 | 88 |
|  |  | Galatheidae | *Galathea* | *Galathea intermedia* | | 3709 | | 2 | 1 | 100 | 90 |
|  |  |  | *Galathea* |  | | 168 | | 2 | 1 | 95 | 90 |
|  |  | Goneplacidae | *Goneplax* | *Goneplax rhomboides* | | 28922 | | 15 | 4 | 99 | 88 |
|  |  |  | *Goneplax* |  | | 201 | | 5 | 2 | 94 | 92 |
|  |  |  |  |  | | 232 | | 6 | 3 | 91 | 91 |
|  |  | Inachidae |  |  | | 382 | | 1 | 1 | 92 | 92 |
|  |  | Paguridae | *Pagurus* | *Anapagurus hyndmanni* | | 135 | | 2 | 1 | 99 | 97 |
|  |  |  | *Pagurus* | *Pagurus bernhardus* | | 222 | | 5 | 1 | 99 | 95 |
|  |  |  | *Pagurus* | *Pagurus prideaux* | | 65 | | 2 | 1 | 99 | 92 |
|  |  |  | *Pagurus* |  | | 84 | | 1 | 1 | 92 | 93 |
|  |  | Pilumnidae | *Pilumnus* | *Pilumnus hirtellus* | | 9535 | | 30 | 2 | 98-99 | 93 |
|  |  |  |  |  | | 42 | | 8 | 2 | 92-93 | 62-92 |
|  |  | Polybiidae | *Liocarcinus* | *Liocarcinus navigator* | | 133 | | 2 | 1 | 99 | 93 |
|  |  |  | *Liocarcinus* |  | | 207 | | 4 | 1 | 94 | 93 |
|  |  |  | *Necora* | *Necora puber* | | 89 | | 1 | 1 | 100 | 100 |
|  |  | Porcellanidae | *Pisidia* | *Pisidia longicornis* | | 253 | | 4 | 1 | 99 | 93 |
|  |  | Portunidae |  |  | | 10983 | | 9 | 2 | 83 | 99 |
|  |  | Processidae | *Processa* | *Processa nouveli holthuisi* | | 93 | | 1 | 1 | 99 | 93 |
|  |  | Sesarmidae |  |  | | 25 | | 1 | 1 | 85 | 100 |
|  |  | Upogebiidae | *Upogebia* | *Upogebia deltaura* | | 526 | | 3 | 1 | 99 | 92 |
|  |  | Xanthidae | *Xantho* | *Xantho pilipes* | | 9 | | 1 | 1 | 97 | 96 |
|  | Diplostraca | Sididae | *Penilia* | *Penilia avirostris* | | 1983 | | 8 | 1 | 99 | 96 |
|  | Euphausiacea | Euphausiidae | *Nyctiphanes* |  | | 1 | | 1 | 1 | 92 | 92 |
|  | Poecilostomatoida | Oncaeidae | *Oncaea* |  | | 31 | | 3 | 1 | 97 | 93 |
| Chaetognatha | Phragmophora | Eukrohniidae |  |  | | 84 | | 2 | 1 | 87 | 95 |
| Chordata | Perciformes | Gobiidae | *Crystallogobius* | *Crystallogobius linearis* | | 29 | | 1 | 1 | 99 | 92 |
| Echinodermata | Euryalida | Euryalidae |  |  | | 9205 | | 9 | 4 | 79-80 | 94-100 |
|  | Ophiurida | Ophiactidae |  |  | | 28 | | 3 | 1 | 83 | 83 |
|  |  | Ophiuridae |  |  | | 2700 | | 5 | 1 | 82 | 91 |
| Cnidaria | Leptothecata | Campanulariidae | *Obelia* | *Obelia geniculata* | | 5 | | 1 | 1 | 99 | 90 |
|  |  |  |  |  | | 29 | | 1 | 1 | 81 | 99 |
|  | Siphonophorae | Diphyidae | *Muggiaea* |  | | 6 | | 1 | 1 | 97 | 98 |
| Mollusca | Architaenioglossa | Viviparidae |  |  | | 61 | | 3 | 1 | 74 | 99 |
|  | Veneroida | Lasaeidae |  |  | | 1331 | | 4 | 1 | 79 | 93 |
|  |  | Montacutidae | *Tellimya* | *Tellimya ferruginosa* | | 85 | | 1 | 1 | 99 | 92 |
| **Phylum** | **Order** | **Family** | **Genus** | **Species** | **Reads** | | **Occ** | | **MOTUs** | **Id** | **QC** |
| ***A. media* COI gene** | | | | | | | | | | | |
| Arthropoda | Calanoida | Clausocalanidae | *Clausocalanus* | *Clausocalanus jobei* | 306 | | 2 | | 2 | 98-99 | 87-92 |
|  |  |  | *Ctenocalanus* | *Ctenocalanus vanus* | 15 | | 2 | | 1 | 99 | 97 |
|  |  |  | *Pseudocalanus* | *Pseudocalanus elongatus* | 235 | | 3 | | 1 | 99 | 92 |
|  |  |  |  |  | 5 | | 2 | | 1 | 94 | 90 |
|  |  | Euchaetidae | *Paraeuchaeta* | *Paraeuchaeta hebes* | 289 | | 1 | | 1 | 99 | 93 |
|  |  | Paracalanidae | *Paracalanus* | *Paracalanus parvus* | 9 | | 1 | | 1 | 98 | 95 |
|  |  |  | *Paracalanus* |  | 31169 | | 10 | | 3 | 94-96 | 91-93 |
|  | Decapoda | Goneplacidae | *Goneplax* | *Goneplax rhomboides* | 4 | | 3 | | 4 | 99 | 88 |
|  |  | Paguridae | *Pagurus* | *Pagurus prideaux* | 97 | | 2 | | 1 | 99 | 92 |
|  |  | Pilumnidae | *Pilumnus* | *Pilumnus hirtellus* | 2 | | 2 | | 2 | 98-99 | 93 |
|  |  | Polybiidae | *Necora* | *Necora puber* | 85 | | 1 | | 1 | 100 | 100 |
|  |  | Porcellanidae | *Pisidia* | *Pisidia longicornis* | 2 | | 2 | | 1 | 99 | 93 |
|  |  | Portunidae |  |  | 1 | | 1 | | 1 | 83 | 99 |
|  | Euphausiacea | Euphausiidae | *Nyctiphanes* |  | 61 | | 4 | | 1 | 92 | 92 |
|  | Poecilostomatoida | Oncaeidae | *Oncaea* |  | 1 | | 1 | | 1 | 97 | 93 |
|  |  |  |  |  | 62 | | 2 | | 2 | 86-87 | 84 |
| Chaetognatha | Phragmophora | Eukrohniidae |  |  | 17 | | 3 | | 1 | 87 | 95 |
| Echinodermata | Euryalida | Euryalidae |  |  | 2530 | | 4 | | 4 | 80 | 94-100 |
|  | Ophiurida | Ophiuridae |  |  | 59 | | 1 | | 1 | 84 | 98 |
|  |  |  |  |  | 1 | | 1 | | 1 | 82 | 91 |
| Cnidaria | Leptothecata | Campanulariidae | *Obelia* | *Obelia geniculata* | 2596 | | 15 | | 1 | 99 | 90 |
|  | Siphonophorae | Diphyidae | *Muggiaea* |  | 32 | | 5 | | 1 | 97 | 98 |
| Nermertina | Heteronemertea | Lineidae | *Cerebratulus* |  | 7 | | 1 | | 1 | 99 | 92 |

| Phylum | Order | Family | Genus | Species | Reads | Occ | MOTUs | Id | QC | |
| --- | --- | --- | --- | --- | --- | --- | --- | --- | --- | --- |
| ***O. vulgaris*16Sa gene** | | | | | | | | | |  |
| Arthropoda | Decapoda | Alpheidae |  |  | 2 | 1 | 1 | 87 | 99 | |
|  |  | Carcinidae | *Carcinus* | *Carcinus maenas* | 56916 | 21 | 2 | 100 | 91 | |
|  |  |  |  |  | 17 | 3 | 1 | 95 | 99 | |
|  |  |  |  |  | 132 | 5 | 3 | 94 | 88 | |
|  |  | Crangonidae | *Crangon* | *Crangon crangon* | 27 | 2 | 1 | 100 | 100 | |
|  |  | Diogenidae |  |  | 550 | 3 | 1 | 80 | 99 | |
|  |  | Galatheidae | *Galathea* | *Galathea squamifera* | 825 | 1 | 1 | 99 | 99 | |
|  |  | Goneplacidae | *Goneplax* | *Goneplax rhomboides* | 2601 | 8 | 1 | 100 | 99 | |
|  |  | Inachidae | *Macropodia* | *Macropodia parva* | 93 | 3 | 1 | 100 | 100 | |
|  |  |  | *Inachus* | *Inachus dorsettensis* | 17 | 2 | 1 | 97 | 99 | |
|  |  |  |  |  | 122 | 5 | 2 | 94 | 99 | |
|  |  |  |  |  | 27 | 1 | 1 | 89 | 99 | |
|  |  | Paguridae | *Pagurus* | *Pagurus prideaux* | 121 | 1 | 1 | 99 | 99 | |
|  |  |  |  | *Pagurus bernhardus* | 428 | 4 | 1 | 100 | 99 | |
|  |  |  |  |  | 34 | 2 | 1 | 86 | 99 | |
|  |  | Pilumnidae | *Pilumnus* | *Pilumnus hirtellus* | 44955 | 43 | 1 | 100 | 99 | |
|  |  |  |  |  | 93 | 10 | 4 | 96 | 90 | |
|  |  |  |  |  | 6 | 4 | 1 | 100 | 63 | |
|  |  |  |  |  | 24 | 3 | 1 | 93 | 99 | |
|  |  | Pirimelidae | *Pirimela* | *Pirimela denticulata* | 2667 | 5 | 1 | 100 | 99 | |
|  |  |  |  |  | 4 | 2 | 1 | 85 | 99 | |
|  |  | Polybiidae | *Necora* | *Necora puber* | 3209 | 7 | 2 | 98 | 99 | |
|  |  |  |  |  | 2 | 1 | 1 | 100 | 80 | |
|  |  |  | *Liocarcinus* | *Liocarcinus navigator* | 3500 | 4 | 2 | 100 | 87 | |
|  |  |  |  |  | 2339 | 3 | 1 | 95 | 99 | |
|  |  |  |  |  | 2358 | 5 | 3 | 93 | 90 | |
|  |  | Porcellanidae | *Pisidia* | *Pisidia longicornis* | 1209 | 6 | 3 | 98 | 99 | |
|  |  | Processidae | *Processa* | *Processa edulis crassipes* | 5 | 2 | 1 | 99 | 99 | |
|  |  | Thiidae | *Thia* | *Thia scutellata* | 4 | 1 | 1 | 100 | 99 | |
|  |  | Upogebiidae |  |  | 812 | 5 | 2 | 90 | 99 | |
|  |  | Varunidae |  |  | 20 | 1 | 1 | 93 | 99 | |
|  | Diplostraca | Podonidae | *Podon* | *Podon intermedius* | 50 | 6 | 1 | 98 | 99 | |
|  | Euphausiacea | Euphausiidae | *Nyctiphanes* | *Nyctiphanes couchii* | 549 | 5 | 2 | 99 | 100 | |
| Echinodermata | Ophiurida | Amphiuridae | *Amphiura* | *Amphiura abyssorum* | 80 | 5 | 1 | 98 | 99 | |
| Mollusca | Mytilida | Mytilidae | *Mytilus* | *Mytilus galloprovincialis* | 19 | 2 | 1 | 98 | 99 | |
| **Phylum** | **Order** | **Family** | **Genus** | **Species** | **Reads** | **Occ** | **MOTUs** | **Id** | **QC** | |
| ***A. media* 16Sa gene** | | | | | | | | | |  |
| Arthropoda | Calanoida | Candaciidae | *Candacia* | *Candacia armata* | 46 | 2 | 1 | 98 | 99 | |
|  | Decapoda | Carcinidae | *Carcinus* | *Carcinus maenas* | 317 | 3 | 2 | 100 | 91 | |
|  |  | Goneplacidae | *Goneplax* | *Goneplax rhomboides* | 14 | 2 | 1 | 100 | 99 | |
|  |  | Paguridae | *Pagurus* | *Pagurus bernhardus* | 8 | 1 | 1 | 100 | 99 | |
|  |  | Pilumnidae | *Pilumnus* | *Pilumnus hirtellus* | 122 | 6 | 1 | 100 | 99 | |
|  |  | Polybiidae | *Necora* | *Necora puber* | 1 | 1 | 2 | 98 | 99 | |
|  |  |  | *Liocarcinus* | *Liocarcinus navigator* | 49 | 1 | 2 | 100 | 87 | |
|  |  | Porcellanidae | *Pisidia* | *Pisidia longicornis* | 231 | 7 | 3 | 98 | 99 | |
|  |  | Upogebiidae |  |  | 35 | 2 | 2 | 90 | 99 | |
|  |  | Varunidae |  |  | 2 | 1 | 1 | 93 | 99 | |
|  | Diplostraca | Podonidae | *Podon* | *Podon intermedius* | 20 | 2 | 1 | 98 | 99 | |
|  | Euphausiacea | Euphausiidae | *Nyctiphanes* | *Nyctiphanes couchii* | 8 | 1 | 2 | 99 | 100 | |
| Echinodermata | Ophiurida | Amphiuridae | *Amphiura* | *Amphiura abyssorum* | 82 | 1 | 1 | 98 | 99 | |
|  |  | Ophiuridae | *Ophiura* | *Ophiura albida* | 10 | 1 | 1 | 98 | 99 | |

| **Phylum** | **Order** | **Family** | **Genus** | **Species** | **Reads** | **Occ** | **MOTUs** | **Id** | **QC** |
| --- | --- | --- | --- | --- | --- | --- | --- | --- | --- |
| ***O. vulgaris* 16Sb gene** | | | | | | | | | |
| Cnidaria | Leptothecata | Prayidae | *Rosacea* | *Rosacea flaccida* | 4 | 2 | 1 | 95 | 98 |
| ***A. media* 16Sb gene** | | | | | | | | | |
| Cnidaria | Leptothecata | Campanulariidae | *Obelia* | *Obelia geniculata* | 32 | 4 | 1 | 99 | 95 |
| Cnidaria | Leptothecata | Prayidae | *Rosacea* | *Rosacea flaccida* | 7 | 3 | 1 | 98 | 95 |
| Chordata | Salpida | Salpidae | *Thalia* | *Thalia democratica* | 3 | 1 | 1 | 99 | 95 |

**Supplementary material 2**

Total number of reads detected in each predator with the different pair of primers (COI, 16Sa and 16Sb), minimum (min), maximum (max), mean and standard error (se). Reads were classified depending on their origin as contamination, predator or potential prey.

|  | | **reads** | | | | | | | | | | | |
| --- | --- | --- | --- | --- | --- | --- | --- | --- | --- | --- | --- | --- | --- |
|  |  | **contamination** | | | | **prey** | | | | **predator** | | | |
| **Predator** | **Primer** | total | min-max | mean | se | total | min-max | mean | se | total | min-  max | mean | se |
| **octopus** | **COI** | 13068 | 0-4253 | 204.19 | 78.38 | 74430 | 0-13251 | 1162.97 | 363.89 | 1641934 | 2154-103790 | 25655.22 | 2763.88 |
|  | **16Sa** | 39 | 0-20 | 0.74 | 0.39 | 121054 | 0-24021 | 2284.04 | 731.93 | 503021 | 1-38641 | 9490.96 | 1247.75 |
|  | **16Sb** | 0 | 0 | 0.00 | 0.00 | 4 | 0-3 | 0.09 | 0.07 | 313 | 0-110 | 7.11 | 3.42 |
| **squid** | **COI** | 21372 | 0-15190 | 667.88 | 482.24 | 37585 | 0 -31884 | 1174.53 | 994.26 | 1299233 | 45-128187 | 40601.03 | 4891.55 |
|  | **16Sa** | 6856 | 0-6833 | 457.07 | 455.42 | 975 | 0-559 | 65.00 | 38.05 | 112343 | 12-26041 | 7489.53 | 1851.61 |
|  | **16Sb** | 87 | 0-66 | 4.83 | 3.69 | 42 | 0-18 | 2.33 | 1.10 | 128661 | 1-29007 | 7147.80 | 1938.37 |
